# Experimental Research of Ratio between Residual and Elastic Strains ε^res^/ε^E^ in High-Strength Concrete Beams Subjected to Bending

**DOI:** 10.3390/ma14206007

**Published:** 2021-10-12

**Authors:** Hydayatullah Bayat, Andrzej Ubysz, Marek Maj, Marek Chalecki, Jarosław Wójt, Ashot Tamrazyan

**Affiliations:** 1 Department of Mechanics and Building Constructions, Institute of Civil Engineering, Warsaw University of Life Sciences (SGGW), Nowoursynowska 159, 02-776 Warsaw, Poland; jaroslaw_wojt@sggw.edu.pl; 2 Institute of Building Engineering, Faculty of Civil Engineering, Wroclaw University of Science and Technology, Wybrzeże Wyspianskiego 27, 50-370 Wrocław, Poland; marek.maj@pwr.edu.pl; 3 Reinforced Concrete and Masonry Structures Department, National Research Moscow State Civil Engineering University, 26, Yaroslavskoye Shosse, 129337 Moscow, Russia; tamrazian@mail.ru

**Keywords:** mechanics of concrete constructions, residual strains, elastic strains

## Abstract

In the mechanics of concrete constructions, one distinguishes between residual and elastic strains. Cyclic loadings evoke changes mainly in the elastic strains; however, the total strains are decided by the sum of the residual and elastic strains. The knowledge of the ratio between the residual and total strains allows to predict the total deformations of a construction assuming it is made from an ideally elastic material. This paper presents the effect of the load level at the values of the residual strains for beams made of high-strength concretes and subjected to bending. The investigations showed that the share of residual strains for these concretes differed from the share of residual strains for concretes of lower classes. The investigations were made for cyclically loaded concrete samples and ferroconcrete beams for various relative load levels. The ratio between the residual and total strains was presented in the form of a dependence on the relative load level of the element. An important conclusion was that, after the crack formation, the share of residual stresses reduced, along with the increase in the concrete strength and elastic features of the construction which started to predominate.

## 1. Introduction

The investigations of the relation between stress and strain (σ−ε) are fundamental and are performed in order to precisely examine the material response to acting loads. In the case of concrete constructions, the detailed evaluation of the relation σ−ε enables the prediction of a behavior of the construction as a dependence on its stress state. In most cases, experimental tests are performed with statically increasing values of an acting force (compressing or tensile), from zero up to the value which destroys a sample. If only the most important quantities characterizing a material are sought, this is usually sufficient. However, if one takes into account that real engineering constructions work under loads varying in different ways (not always statically) and that the loading–unloading cycles can repeat many times, then the investigations of the relation σ−ε should also be expanded by the aforementioned factors. In this regard, the constitutive material models should be created which consider the most important factors responsible for a material’s behavior.

The first studies concerning this topic can be found in [1]; other relevant publications are [2,3,4], where samples made of an ordinary concrete subjected to cyclic compression were investigated. Karsan and Jirsa [3] noticed that the behavior of the concrete during cyclic loading was related to a previous value of loads. Moreover, they noticed that it was possible to credibly assess the number of cycles until destruction. A vast majority of the developed models were based on the compressed samples; however, the studies concerning the behavior of ordinary concretes under tension with consideration of the loading–unloading cycles can be also found [5,6].

The constitutive models of concrete, presented in the literature, are usually based on the theories of elasticity, plasticity or fracture mechanics. This latter approach is a good reflection of the material behavior but requires the determination of a large number of parameters which is cumbersome and limits the application of such models in the engineering practice; several works within this scope are accessible where a good agreement between the developed models and the experimental results was achieved [5,6,7]. Therefore, a certain modification is presented in [6]; this work contains formulas which aim to ensure a good compatibility with a nonlinear analysis made using the Finite Element Method. The models based on the smeared crack approach are also developed [8,9,10,11].

When analyzing the aforementioned publications, one can note a distinct and continuous striving to upgrade the models and increase their rational applicability in an engineering practice. An important summary in this aspect was made by Aslani and Jowkarmeimandi [12]; their developed model is elaborated in Section 2.2.

Along with the improvement of the research methods and constitutive models, a distinct progress in the widely understood concrete technology occurs in building construction as well. Due to this, high-strength concretes become increasingly common; according to the currently applied criteria, this designation denotes a material with a characteristic compressive strength of over 50 MPa. The range of this strength is wide and amounts to ca. 100 MPa. The concrete with such high-strength parameters provides huge possibilities for the design of engineering constructions. The research works concerning the development of the appropriate constitutive models describing the high-strength concrete can be found in the literature. Aslani and Nejadi [13] proposed a constitutive model for a high-strength concrete subjected to monotonic and cyclic loads. The model considered a transversal reinforcement of a very high and “normal” strength. It was related to the application of such constructions in seismically active regions where horizontal loads was very important from the point of view of construction durability and safety. As the authors announced, a good concordance of the model to the experimental results was examined.

Rashid et al. [14] presented the important engineering properties of an ordinary concrete for a wide range of compressive strengths, especially the impact of the concrete strength on the elasticity modulus, tensile strength and Poisson’s ratio of concrete. The adequacy of some of the known relationships for predicting the elasticity modulus and tensile strength of the concrete was examined and suitable expressions were proposed to cover a concrete strength of up to 120 MPa.

The stress–strain relation is not, in all cases, a basic topic of the experimental investigations. The possibility of the credible prediction of the strains of components in a construction allows for their adaptation to the aesthetic and usability requirements resulting from its purpose. The advanced (nonlinear) Finite Element Method meets such expectations [8,10,15,16,17,18,19]. Nevertheless, it is worth seeking alternative methods of assessment for the total strains of the construction depending on the value of the load, as well as the way in which it applies to and acts on the construction.

In high-strength concretes, a broader elastic range is observed in the relation σ−ε than in classical concretes. This paper presents how to experimentally separate the elastic and plastic part of a strain in a ferroconcrete beam subjected to bending. The results of the investigations presented below align with the subject undertaken in a previous work [20]. This analysis is based on the current Borcz model [21] which emulates the linear elastic–plastic behavior of concrete. The experiments described in the present paper were performed in order to examine the share of the residual strains for multiple loading–unloading cycles of a high-strength concrete beam. The investigations were intended as a verification of a model based on an experimental determination of the values of coefficients describing elastic and permanent deformations. These values were already partially calculated for ordinary concretes [22], whereas the coefficients for high-strength concretes were determined experimentally. The verification of the share of residual and elastic strains in the high-strength concrete allows designers to predict the real stresses and strains based on calculations made using a program which emulates a linear elastic model of the concrete. Moreover, such a calculational model allows the consideration of rheological deformations (mainly creep) occurring under long-lasting loads.

Many numerical models were developed in the literature, describing the nonlinear behavior of the concrete and using, i.a., the environments Abaqus [8,17], LS-Dyna [23], *fib* Model Code 2010 [24], Adaptic [25] as well as RC-FINEL, Ansys, Strains. These models were mostly described by constitutive equations and the fundamentals of these models were based on experimental investigations [8,9]. Due to the high possibilities of the nonlinear finite element method (FEM) analysis, the range of undertaken issues was very wide; the works by Castaldo et al. [15,16] and Teng et al. [23] are relevant examples in this matter. The development of such models is still in progress due to the increasing interest in the application of concrete for designing constructions in various environmental conditions (e.g., seismic and thermal loads, and corrosive processes), as well as for analysis using advanced computer programs. Initially, the concrete models were limited to a description of the physical features of the concrete during a uniaxial and monotonous compression and unloading. In recent years, the aforementioned concrete models occurred which, applied in the advanced programs, allowed for the analysis of the strains and stresses of cyclic compressing and tensile loads in the material yield zones [12,20,21]. The development of a model describing a complex structure of the concrete which shows the cyclic relations σ−ε in pre- and post-critical states, is expected. It is obvious that a perfect model of the concrete in a FEM system should consider the physical features of the concrete before and after the crack formation which requires the gathering of a large amount of experimental data. Therefore, there is a need to develop simpler models of the concrete which could describe its features with the use of more basic parameters such as the ratio between the residual and total strains under multiple loading and unloading.

The paper presents the influence of a load level on the values of the residual strains for high-strength concrete beams subjected to bending. The aim of the investigation was to present, in the case of an experiment, how the share of the residual strains in relation to the elastic strains changed for different levels of the load acting on the construction. The studies so far undertaken concerned the mainly compressed elements. In the case of the bent elements, the behavior after the first cracks occurred in the construction was also important (usually it referred to the serviceability limit state). The experimental investigations made for the compressed elements showed that the share of the residual strains for the high-strength concretes differed from the share of residual strains for concretes of lower classes [20,21,22]. Such information should be considered during the formulation of constitutive models used in the design of ferroconcrete and prestressed constructions. It was experimentally presented in the described investigations how the share of residual stresses in an element subjected to bending changed with the increase in the concrete strength. Therefore, it supplements the results of the investigations performed for the elements subjected exclusively to stretching.

## 2. Numerical Quasi-Linear Models of the Concrete

### 2.1. Example Models of the Concrete in the Literature

The analysis of the relation σ−ε for concrete elements under multiple loading and unloading presents a good concordance between the calculational models and experiments. An important element of the analysis is the states in which plastic deformations of the material occur. Such a level of the deformations where a construction deflects significantly, much more than under an initial load, can be observed both in the regions prone to earthquakes and in cyclically loaded constructions, as well as in rheological processes. There are many examples in the literature concerning investigations concerning a description of the behavior of high-strength concretes under loads of all kinds, e.g., fatigue [26,27], monotonous [28] or cyclic, and the creation of an unconfined and confined model of the concrete. Among them, one can specify models of non-reinforced and reinforced concretes under cyclic loads [3,12,25,29,30,31,32,33,34,35].

**Figure 1:**
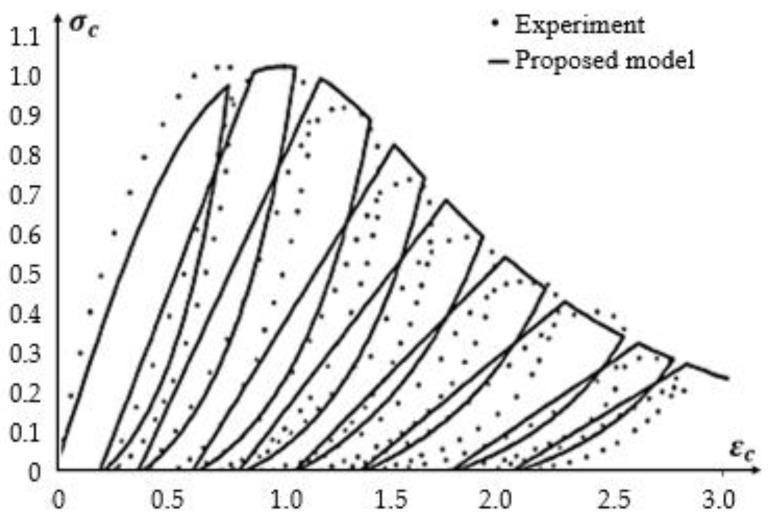
Relation σ−ε for a compressed element according to experimental results and a theoretical model. Adapted from Ref. [1].

Constitutive equations are applied for a description of the stress–strain relation in the concrete. Aslani and Jowkarmeimandi [12] described this relation in a compression and tension zone, as well as the cracking effect caused by the material (concrete) fatigue in the tension zone due to a cyclic load, and compared the developed models to the results of the experiments performed by Sinha et al. [1] and other authors. The description in the compression zone under cyclic loads takes into account a modification of the model of Carreira and Chu [36] as well as a nonlinear description of the concrete behavior during unloading with use of a power-type function. Sima et al. [11] also described a constitutive model of the concrete for cyclic compressing and tensile loads; however, this model contains an improvement based on the “smeared crack” approach. The authors obtained results similar to those presented in Figure 1.

Based on the experimental results, Bahn and Hsu [2] described the relation σ−ε for a partial loading and unloading with the use of a polynomial function. However, the plastic deformations in the tension zone are described with use of a dependence ε*_pl_* = 0.725ε*_un_* (ε*_un_*—unloading concrete strain) and the influence of a crack formation in the tension zone for a monolithic construction is controlled with the use of a stress value σ*_f_* = *f_c_*/10 which corresponds to a full closing of the crack. The value depends mainly on the concrete strength and the kind of crack.

Von der Haar and Marx [37] described a strain model of a concrete subjected to fatigue loads with consideration of the elastic, viscous, thermal and damage-caused strains. Particular parts of the model were treated as developing individually in accordance with a number of load cycles, the duration of the tests and the parameters of the cyclic load.

**Figure 2:**
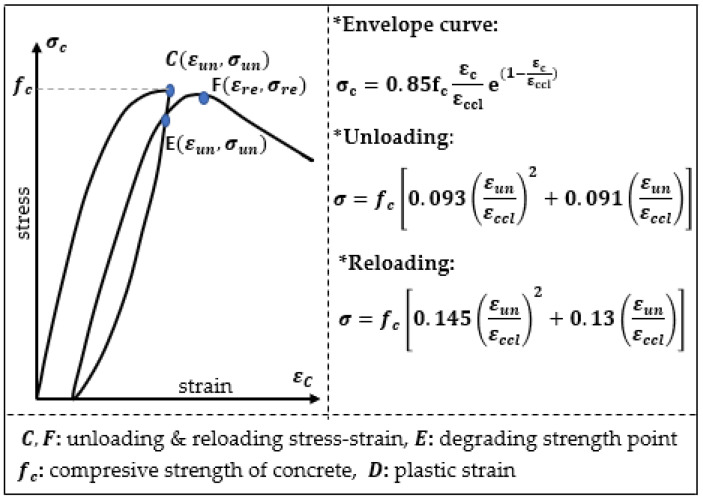
Concrete model according Karsan and Jirsa [3].

There were more models in the literature considering the relation σ−ε during cyclic loading and unloading in a parabolic way, e.g., according to the model of Karsan and Jirsa [3] the unloading relation obtained from the cyclic compression of a reinforced concrete was described by a second-order parabolic curve (Figure 2).

**Figure 3:**
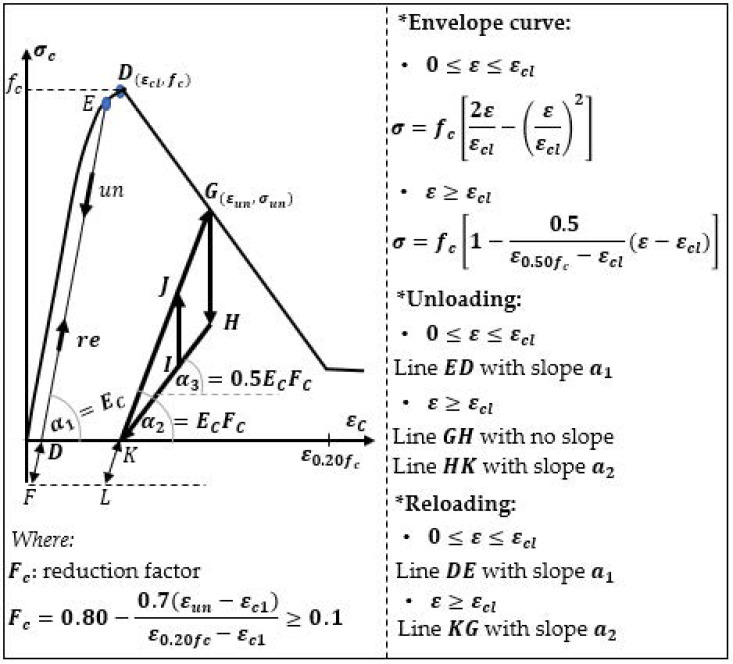
Concrete model according Blakeley and Park [29].

Blakeley and Park [29] applied a simplified concrete model where the relation σ−ε for loading and unloading was assumed as linear and, for the strains smaller or equal to the strain corresponding to the peak stress ε_c_ ≤ ε_cl_, did not consider energy dissipation or stiffness reduction, as well as the effect of confinement (Figure 3). The stiffness reduction was considered after exceeding the point D(ε_cl_, *f_c_*) by a reduction in the concrete’s compressive strength. In this model, the partial loading and unloading (lines IJ-JG and GH-HI), as well as the influence of the tension before the crack formation, (lines KL and DF) were described as linear.

**Figure 4:**
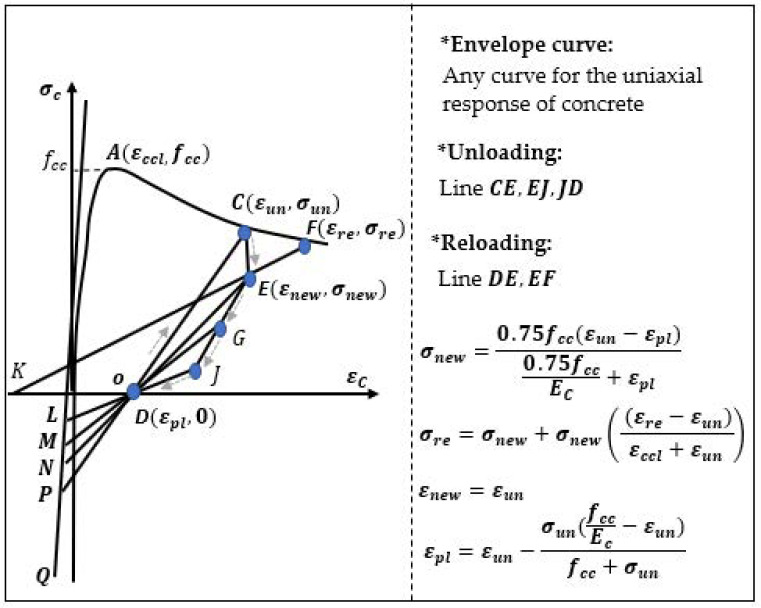
Concrete model according Yankelevsky and Reinhard [30].

A piecewise linear model of the relation σ−ε during cyclic loading and unloading was described by Yankelevsky and Reinhard [30]. The stiffness reduction and yield limit were taken into consideration for the loading–unloading cycles with a piecewise linear slope angle (Figure 4).

**Figure 5:**
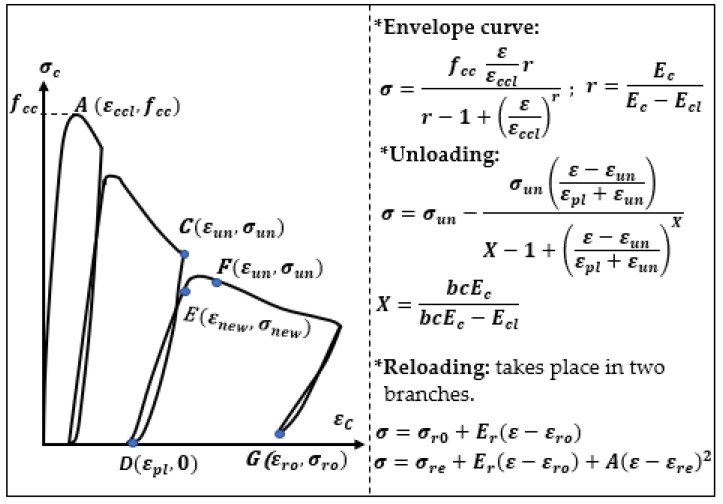
Concrete model according Mander et al. [31].

A concrete model proposed by Mander et al. [31] described to some extent tensile stresses and considered the influence of a reinforcement. This model was a simplified version of the model of Karsan and Jirsa [3]. In this model, the unloading relation was also described by a second-order parabolic curve (Figure 5).

**Figure 6:**
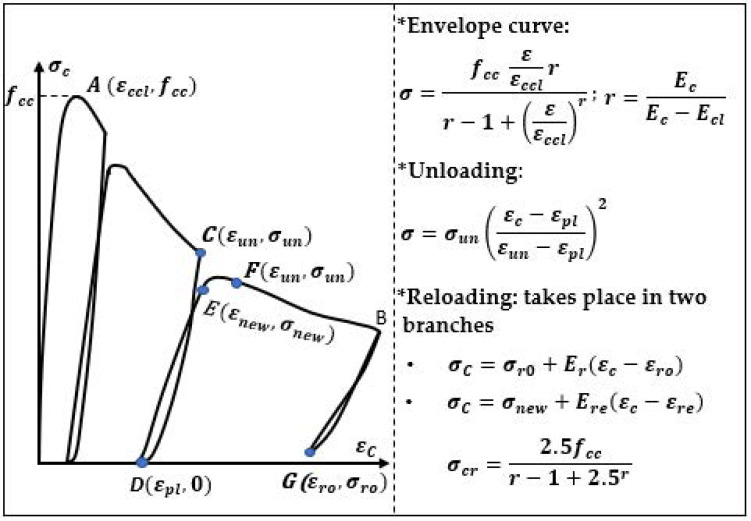
Concrete model according Martinez-Rueda [32].

With the aim of analyzing the displacements to an appropriate extent, as well as to enable a numerical analysis, Martinez-Rueda [32] proposed a modification to Mander’s model. In the Martinez-Rueda model, three different definitions corresponding to a low, medium and high range of strains were applied for the description of plastic strains (Figure 6).

**Figure 7:**
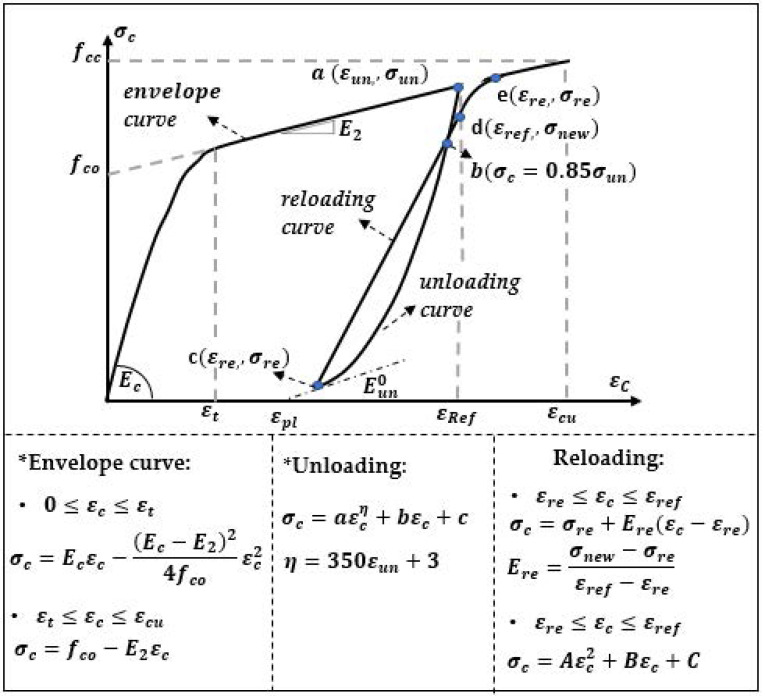
Concrete model according Lam et al. [33].

Lam et al. [33] described the relation σ−ε for a uniaxial compression. The loading in this model was described piecewise linearly and parabolically, whereas the unloading was only described parabolically (Figure 7).

**Figure 8:**
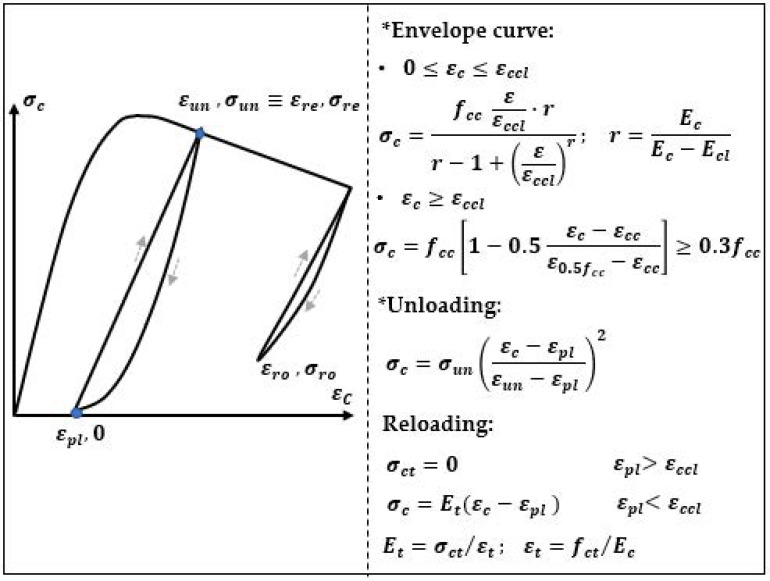
Concrete model according Konstantinidis et al. [25,38].

Konstantinidis et al. [25] proposed a concrete model with the possibility of a numerical analysis with the use of the program, Adaptic. The authors extended the models of Kappos and Konstantinidis [38] for a description of a high-strength concrete. In this extended model, the relation σ−ε was assumed as linear during the loading and parabolic of the second-order during unloading (Figure 8).

Sadeghi et al. [34] presented a constitutive law for a numerical simulation of the behavior of a reinforced concrete with the use of a uniaxial relation σ−ε for the description of a loading–unloading cycle. The increase in plastic strains during the loading and unloading with a consideration of transversal reinforcement was described in this model with the use of a parabolic curve.

A constitutive model of a compressed concrete proposed by Breccolotti et al. [35], based on the model of Sima et al. [11], described the cycles of stresses for a constant and variable amplitude. The loading and unloading of this model were described by linear and parabolic functions. This model could be used for the analysis of fatigue loads.

A detailed explanation of the models of Sadeghi [34], Breccolotti [35] and the other abovementioned models can be found in the relevant literature provided at the end of this paper.

The high-strength concretes differed in terms of physical features from the concretes commonly applied in 20th century. They concerned not only such basic parameters as characteristic strength or Young’s modulus but also the plastic features, elongation, fatigue properties, cooperation between the concrete and reinforcement, and the σ−ε curve for loading and unloading, etc. In the presented models, the authors analyzed each of these parameters. For practical calculations, the models are often sought which allows for a simple description of the most important features and relations in the concrete. Below it is presented in the Borcz model [21], describing the relation σ−ε in the concrete with the use of linear functions, as well as the verification of this model.

### 2.2. Linear Elastic–Plastic Borcz Model 

**Figure 9:**
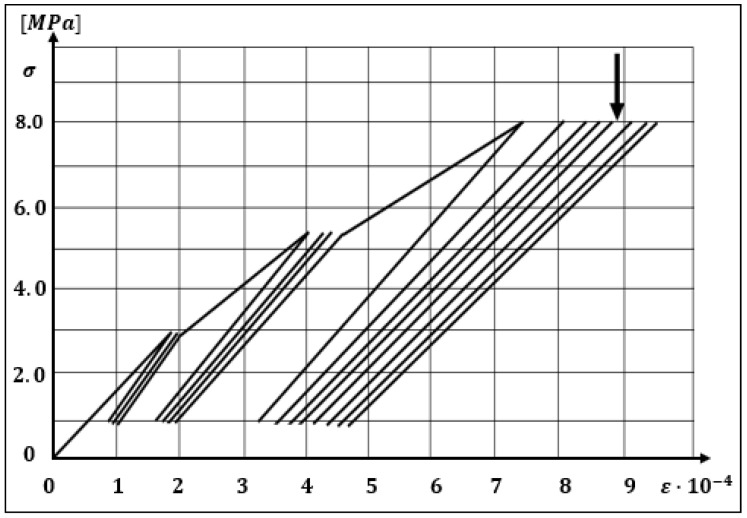
Relation σ−ε according to Borcz [21].

The model [21] describes the cyclic relations σ−ε in a pre-critical and post-critical range (Figure 9). The suggestion of the model is an assumption that, during cyclic loads, the repeatable cycles are quasi-linear and a dependence exists between the residual and total strains for various levels of loads. The separation of residual and elastic strains simplifies the static calculations and allows the design of constructions with use of simple programs using the linear elastic material model.

The basic assumptions of the Borcz theory are as follows:

- The static problem is reduced to a one-dimensional issue.
- If a beam has a constant second area moment, then a reference line covers the line of gravity at the center of the beam cross-section.
- If a beam has a variable stiffness, then the line of gravity at the center of the beam cross-section is not straight. In such a case, it is assumed that the longitudinal coordinates for the whole beam are measured on the one, straight reference line.
- A beam with a variable second-area moment is replaced by a beam with a piecewise constant second-area moment. The beam is divided into sections, of which each has a constant equivalent stiffness.
- It is assumed in this model that the directions of principal stresses during loading are constant.
- For repeatable loads, the model assumes the total reversibility of the elastic part of the strain after unloading a construction.
- The total strain tensor ε*_ij_* in this model is described by a relation:

(1) $$\varepsilon_{ij}=\varepsilon_{ij}^{E}+\varepsilon_{ij}^{res},$$

where $\varepsilon_{ij}^{E}$ is the elastic part of the strain tensor and $\varepsilon_{ij}^{res}$ is the irreversible part of the strain tensor.

This theory was experimentally verified [20,21,39,40]. The obtained results confirmed the adopted assumptions and justified the application of simple calculational models.

The investigations to date primarily concerned a compressive load; no investigations concerning bent elements were found. Such investigations had a great practical meaning if the serviceability limit states were taken into consideration and cracks occurred in a beam subjected to bending. The studies so far undertaken concerning the ratio between the residual and elastic strains for concretes of lower classes showed that this ratio was not constant. There was no information in the literature on how to describe the ratio $\varepsilon_{ij}^{res}/\varepsilon_{ij}^{E}$ for various levels of material effort. The experimental tests provided results proving that the shape of the function described the relation between the ratio $\varepsilon_{ij}^{res}/\varepsilon_{ij}^{E}$ and the load level changes. This information was crucial for the process of the formulation of constitutive models for the computer programs dedicated to the calculations of ferroconcrete and concrete constructions, for the phase after the time instant in which the bending moment is exceeding the value causing cracks. Simultaneously, these investigations was an experimental extension of the Borcz theory which separated the residual and elastic strains in the constitutive model of concrete.

## 3. Investigations

With the aim of obtaining experimental results which showed the relation between the ratio $\varepsilon_{ij}^{res}/\varepsilon_{ij}^{E}$ and the load level for the high-strength concretes under various load levels, seven series of tests were performed of one-span ferroconcrete beams made of concretes with the characteristic strength *f_ck,cube_* = 77.5 MPa (average guaranteed compressive strength according to the standard [41]) and the steel BSt 500. The dimensions of the cross section of each of the beams were 0.20 m × 0.12 m, and the lengths were 1.80 m. In each series three beams were tested, differing in reinforcement ratio, diameter of main bar, type of the bars and duration of the loading.

**Figure 10:**
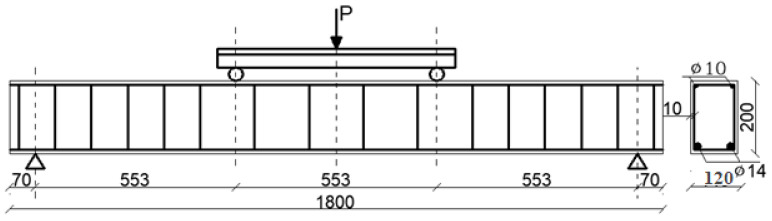
Static schemes and geometry of the beams of the V-th series (all dimensions in mm).

**Figure 11:**
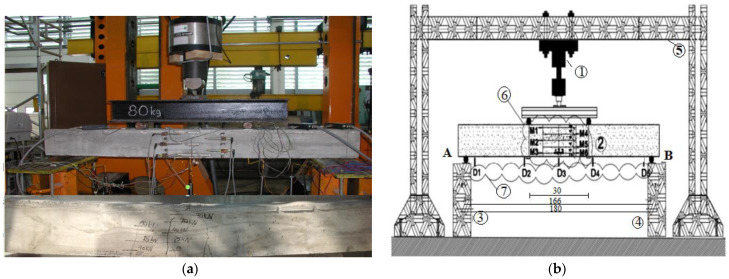
Scheme (**b**) of the test rig (**a**) for the beam of V-th series: 1—actuator, 2—the tested element, 3—roller support, 4—hinged support, 5—main supporting frame, 6—induction sensors measuring horizontal displacement, 7—induction sensors measuring deflection.

The beams were loaded with two concentrated forces according to the scheme presented in Figure 10. The beam was placed on two supports: a roller (A) and a hinge (B, Figure 11). The distance between the concentrated forces and the supports was equal to one-third of the span between supports. The investigations were performed at a test rig (Figure 11) and deflections were forced cyclically with a constant velocity (both during the increase and decrease in the deflections).

**Figure 12:**
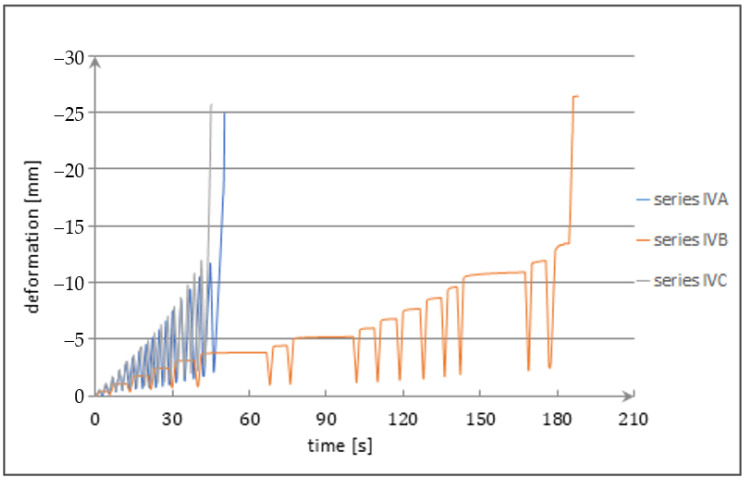
Cycles of strain force in the investigated beam.

The strains and deflections were registered on the induction and resistance sensors. The loads were applied in a continuous and interrupted way. The loading cycle during the investigations is presented in Figure 12.

When the deflections were forced in a continuous and interrupted way, they enabled the registration of short-term rheological effects and the strains remaining after unloading [39]. The layout of the sensors allowed strains to be registered in the concrete through the whole height of the cross-section across the middle of the beam span. The induction sensors (6 pcs) were distributed in a longitudinal section (base) of 300 mm (150 mm left and right of the middle of the span, Figure 11). They registered the horizontal displacements of the base in the tension and compression zone, as well as near the neutral axis.

## 4. Results of Investigations

Seven series of tests on one-span ferroconcrete beams for the reinforcement ratios *r* = 〈0.5%, 1.5%〉 were performed. The good quality concordance between the results showing the ratio $\varepsilon_{ij}^{res}/\varepsilon_{ij}^{E}$ for various levels of material effort were obtained. As the most representative, the results for the series IV and V were chosen.

**Figure 13:**
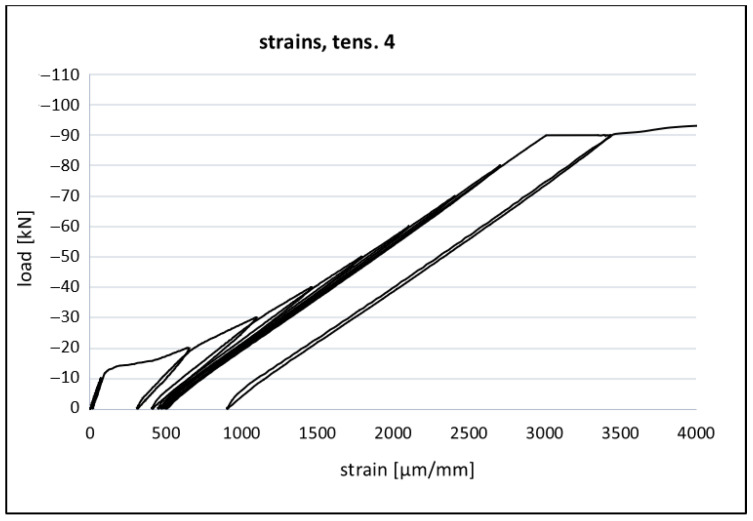
Experimental relationship between strain and load of a reinforcing bar (beam of the series Vc—tensometer N° 4 on the bar).

**Figure 14:**
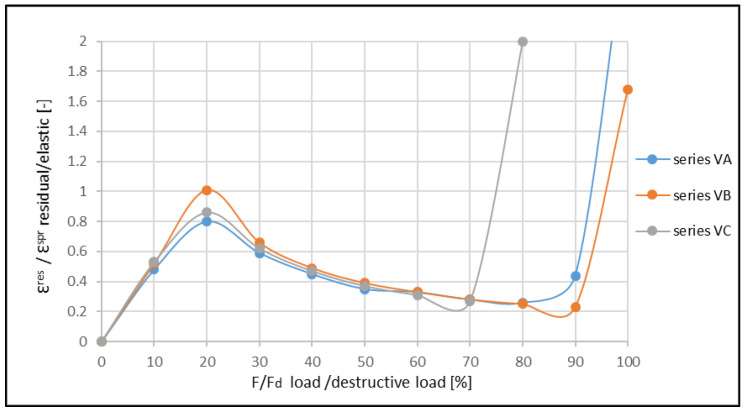
Example graph showing the ratio between residual and elastic strains regarding the ratio between the applied load and destructive load for the beams of V-th series.

Figure 13 and Figure 14 present an example relation between the load and strain in a reinforcing bar. In the initial phase of loading it was observed that a linear relation between load and strain. It proves a good bond between steel and concrete in this phase. In the case of the beams being tested, the first cracks were observed for the loads from the range from 15–20 kN. The strain values registered in the sensors at the reinforcing bars were concordant to the concrete strains. As the load increased, a disturbance of the bond between the concrete and reinforcement was observed. As a result of this disturbance, the increase in strains accelerated. The independent investigations of the reinforcing bars allowed for the conclusion that the increase in strains did not result from a yield in steel but only from a reduction in the equivalent stiffness of the steel and the concrete cross-section area. This phenomenon could also be interpreted as the beginning of the crack formation in the concrete in the tension zone. The further forcing of the strains evoked the subsequent cracks and further increased the strains until the beam was damaged in the cross section.

**Figure 15:**
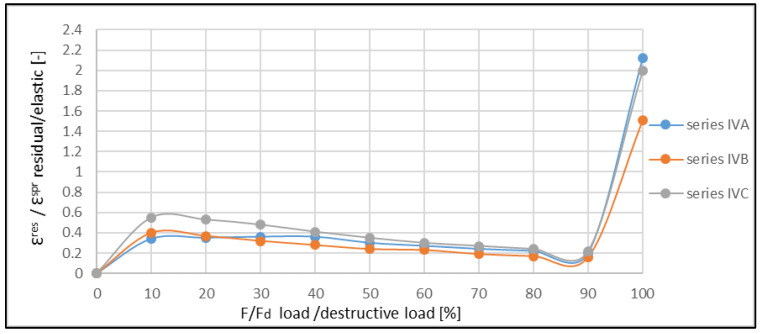
Example graph showing the ratio between residual and elastic strains regarding the ratio between the applied load and destructive load (beams of IV-th series).

**Figure 16:**
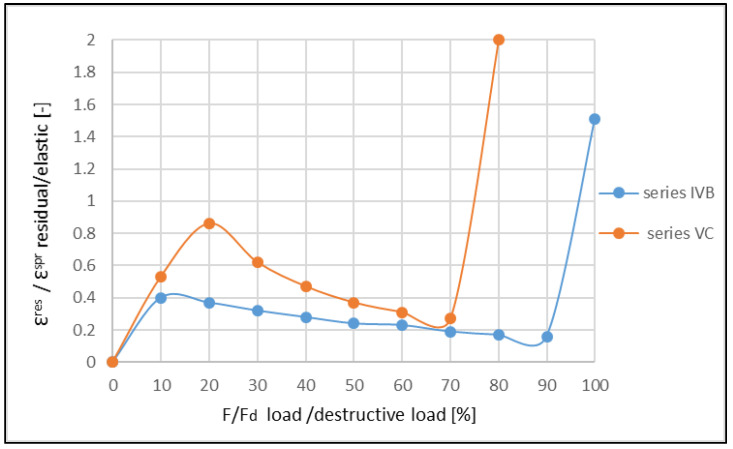
Comparison of the results, showing the ratio between residual and elastic strains for the beams of IV-th and V-th series.

**Figure 17:**
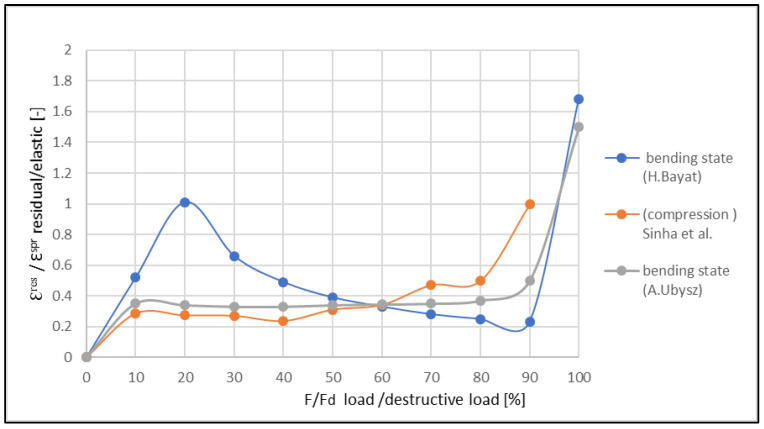
Comparison of the results from the literature [1,20,22] with those obtained by the authors.

The ratio between the residual and elastic strains increased until the moment of the formation of the first crack, then the ratio decreased. Next, the increase in the ratio ε^res^/ε^E^ was observed in a critical phase. Such a variability of this ratio was also confirmed for other reinforcement ratios of the beams (Figure 15 and Figure 16). A comparison of the obtained results with those obtained by Aslani and Jowkarmeimandi [12] and Sinha et al. [1] for the concretes of significantly lower class suggested that this relation changed its character (Figure 17). Some concordance was obtained for the initial phase before the crack formation and for the phase before the damage.

## 5. Discussion of Results

The relations σ−ε for the high-strength concretes show that this material presents more elastic features than the concretes with a lower strength. This concerns both lower loads (where the loading curve has much weaker curvatures and its character is close to the linear one), and higher loads (where the strain increments are small and the destruction is an abrupt and brittle failure) without any long section of strains. In this respect, the shape (but not values) of the graph resembles the graph for the relation σ−ε for a steel with a small elongation.

In the investigations presented above [3,12,25,29,30,31,32,33,34,35], the authors describe the loading curves and, particularly, the unloading curves with the use of parabolic or exponential functions. These functions properly describe the stress–strain relation for the specific investigations; however, the coefficients in these functions depend on the velocity of strain increments. The slower the loading–unloading cycles are, then the more similar to a straight line the curves describing these cycles become. An increment of rheological strains evoked by creep is also a straight line. Due to this, for cyclic loads in a small range, as well as for a description of creep, the double-linear model proposed by Borcz (Figure 9) appears to be justified.

The tangent of the slope angle for the dependence describing the loading and unloading, with the use of the relation σ−ε for various load levels, is almost constant. Its value approximately corresponds with the value of the elasticity modulus. The creep process is described by a horizontal line, whereas the cyclic loading and unloading could be qualified as creep (more precisely, it could be named as “vibrocreep”). Such description of the phenomenon is partially confirmed by Martinez-Rueda [32] and Lam et al. [33]; however, they limit the interpretation of the relation σ−ε to the loading process.

The investigations performed by the authors of this paper confirm the results obtained by Bahn and Hsu [2], who approximate the values of plastic strains in the tension zone with the use of a dependence ε*_pl_* = 0.725 ε*_un_* (ε*_un_*—unloading concrete strain) and the influence of a crack formation in the tension zone for a monolithic construction with the use of a stress value σ*_f_* = *f_c_*/10.

The investigations also allow the establishment of the ratio between the residual and elastic strain in the range of the short-term load. It is clearly visible that the crack formation affects changes in this regard. Before the crack formation, this ratio increases quasi-linearly and approximately reaches the values within the range 〈0.6, 1.0〉. Then, after the crack formation, it slowly decreases down to the level where the material begins to damage. In this respect, the high-strength concrete presents a higher share of the residual strains than the concretes with a lower strength, where the ratio ε^res^/ε^E^ does not exceed the value of 0.5 [22,42].

The differences in the character of the functions showing the ratios ε^res^/ε^E^ for various levels of material effort are clearly visible in Figure 17. For concretes of lower classes, similar characteristics of the function for compressed (cf. [1]) and bent (cf. [20,22]) elements are observed. The clearly descending trend of the graph after the crack formation illustrates the small plastic deformations in the concrete structure in this phase.

## 6. Conclusions

The investigations of the ratio ε^res^/ε^E^ allow for a fast prediction of the expected strains (and deflections) of ferroconcrete elements. However, the potential importance of the concrete class must be emphasized. For the high-strength concretes, the influence of the residual strains after the crack formation is reduced, contrary to the concretes of lower classes, where the ratio ε^res^/ε^E^ is almost constant. Based on the performed experimental investigations, it can be concluded that after crack formation the share of the residual stresses reduces along with the increase in the concrete strength, and the elastic features of the construction start to predominate. Moreover, the ratio ε^res^/ε^E^ is significantly higher for the high-strength concretes than for the concretes with a lower strength.

The experimental investigations undertaken in this paper aim to verify the Borcz theory and show the advantages of this simplified method for the calculation of strains. One can distinguish the following benefits:

1. The development of computational methods based on linear elastic models is justified by the speed and ease of calculations.
2. The approximate results of the experimental investigations from the linear elastic models have an accuracy of up to 5% which is sufficient for the design of most engineering constructions.
3. These models allow the prediction of a strain state in pre-critical states, including accidental loads (earthquake, hurricanes).

The next conclusion from the presented investigations concerns an experimental determination of the ratio ε^res^/ε^E^. The knowledge of the share of residual and elastic strains allows a determination of the internal forces in a construction and its deflections based on the calculations made with the use of the methods applied for constructions made from elastic materials. The results, obtained in such a way, can be approximately corrected allowing for the calculation of the residual and elastic strains in the construction as well as an assessment of the influence of these strains on the total deflections.

The experimental investigation of these relations can be acknowledged as a valuable method for a fast determination of the expected strains, also in the case of more complex ferroconcrete structures. The potential to predict the total strains of the elements or whole constructions without performing very complicated numerical calculations allows for a more reliable design of structures [43] and the acceleration of the design process, especially in the initial (conceptual) stage when the designer chooses the economically and technically optimal static scheme of a construction.

## Data Availability

The data presented in this study are available on request from the corresponding author.
